# Supplementary material for: Mosquito midgut stem cell cellular defense response limits Plasmodium parasite infection
Source: Nat Commun. 2024 Feb 16;15:1422. doi: 10.1038/s41467-024-45550-2 (PMC10873411; doi:10.1038/s41467-024-45550-2)
Supplement: Supplementary file 1 — Supplementary Information [file 41467_2024_45550_MOESM1_ESM.pdf]

Supplementary Materials for

Mosquito midgut stem cell cellular defense response limits Plasmodium parasite infection

Authors: Ana-Beatriz F. Barletta*, Jamie Smith, Emily Burkhart, Simon Bondarenko, Igor V. Sharakhov, Frank Criscione, David O'Brochta, and Carolina Barillas-Mury*
*Corresponding author: cbarillas@niaid.nih.gov and anabeatriz.barlettaferreira@nih.gov

The PDF file includes:

Supplementary Figs. 1 to 8
Supplementary Table 1
Captions Supplementary Movies 1 to 4
Supplementary Movies 1 to 4

Movie S1.

Midgut progenitors in direct contact eliminate *P.berghei* oocysts from the midgut epithelia 10 days after infection (Top view).

Movie S2.

Midgut progenitors in direct contact eliminate *P.berghei* oocysts from the midgut epithelia 10 days after infection (Side view).

Movie S3.

Midgut progenitors in direct contact eliminate *P.berghei* oocysts from the midgut epithelia 14 days after infection (Top view).

Movie S4.

Midgut progenitors in direct contact eliminate *P.berghei* oocysts from the midgut epithelia 14 days after infection (Side view).

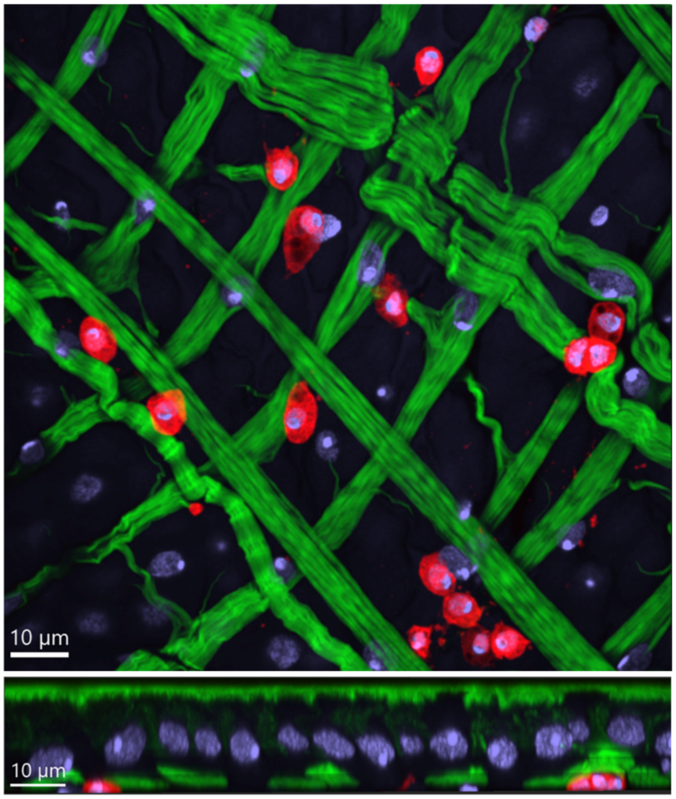

Supplementary Fig. 1. An *Anopheles stephensi* SDA-500 transgenic line (HP10) that expresses a fluorescent reporter (td-Tomato) also in hemocytes. XY and lateral views of hemocytes attached to the midgut surface. Hemocytes are located on top of the muscle layer showed in green. Hemocytes in red, nuclei in blue and actin in green. Scale bar: 10 µm. Micrographs are representative of experiments that were independently reproduced at least 2 times with multiple individuals in each experimental group and obtained similar results.

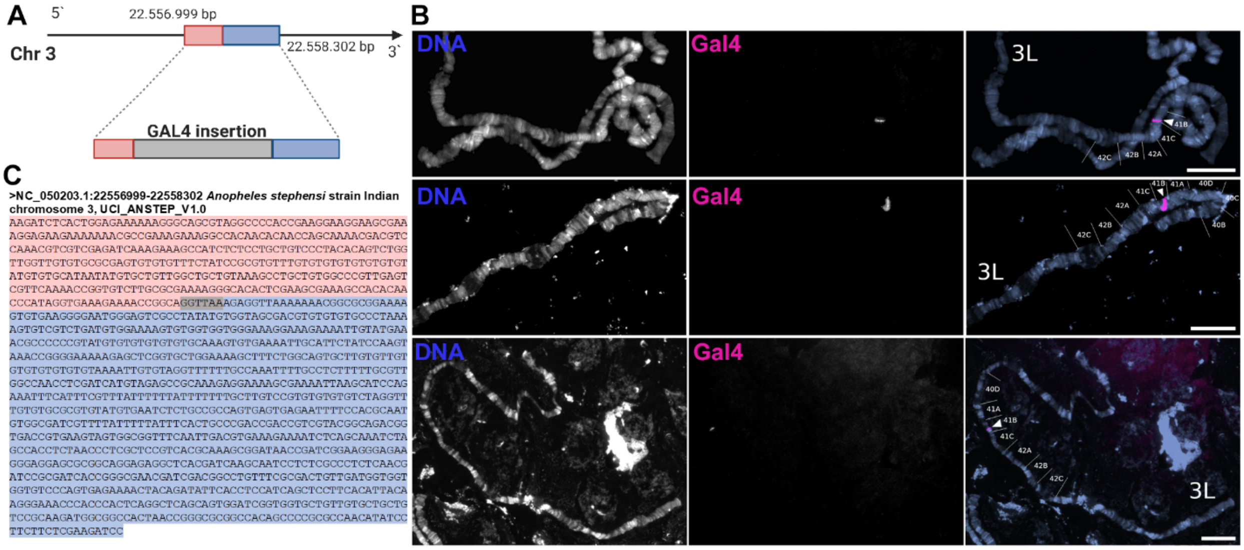

Supplementary Fig. 2. A single GAL4 enhancer-trap insertion was mapped to chromosome 3. (A) Schematic drawing representing the insertion site of the GAL4 enhancer trap in the HP10 transgenic line. (B) FISH to detect GAL4 with ovarian nurse cell polytene chromosomes. A single GAL4 insertion is shown by arrows on chromosome 3 (3L arm; subdivision 41B). First column indicated DNA staining; middle column shows the GAL4 insertion, and the last column is a merged image. Scale bar: 20 µm. Micrographs are representative of experiments that were independently reproduced at least 2 times with multiple individuals in each experimental group and obtained similar results.(C) Region on the genome where the *piggyBac* insertion happened. Portion belongs to chromosome 3 and is located between 22.556.999 bp and 22.558.302 bp. The region highlighted in pink is the 5' prime in blue is the 3' prime flanking regions to the insertion. Nucleotides highlighted in gray is where the sequences mapped overlap and therefore is the potential insertion site.

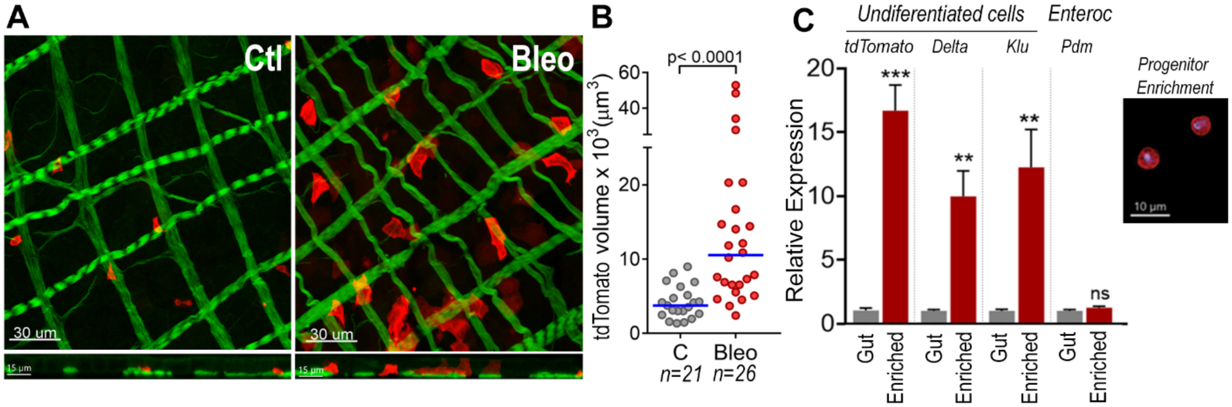

Supplementary Fig. 3. Td-tomato+ midgut cells from *An.stephensi* HP10 transgenic line are epithelial progenitors (stem cells and enteroblasts). (A) Midgut surface of mosquitoes treated with control sugar solution or 25µg/ml bleomycin solution for 48 hours. Actin muscle layer is in green, and progenitors are in red. Scale bar: 30 µm. Micrographs are representative of experiments that were independently reproduced at least 2 times with multiple individuals in each experimental group and obtained similar results. (B) Volume of Td-tomato (td-Tomato+ cells) in control sugar-fed and bleomycin-treated midguts, 2 days post-treatment. Each dot represents an individual midgut. Median is shown as a blue horizontal bar. Mann Whitney U test. (C) Gene expression of td-Tomato, *Delta*, *Klumpfuss* and *Pdm* in whole sugar-fed midguts and fractions enriched for midgut progenitors. Error bars represent mean ± SEM of midgut pools. Unpaired t-test, **P≤0.01, ***P≤0.001. The numerical data underlying the plots in the manuscript are provided as a Source Data File.

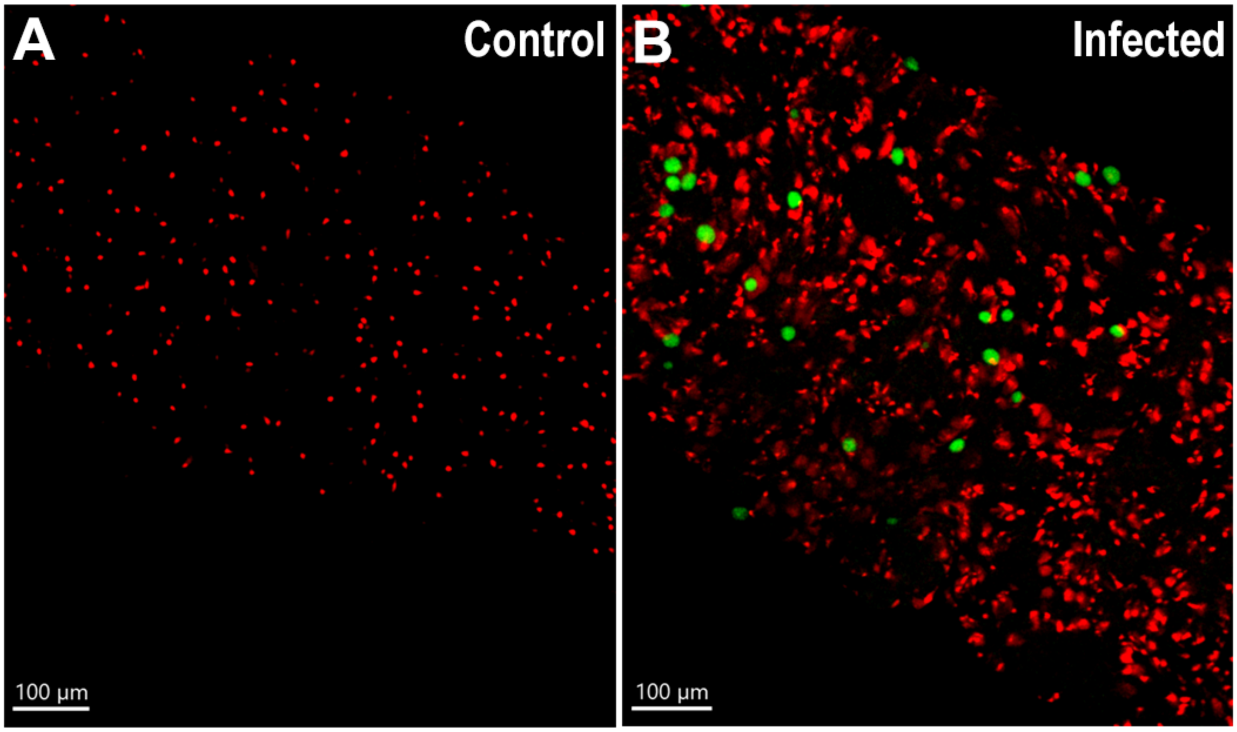

Supplementary Fig. 4. Midgut progenitors proliferate more in infected midguts and form clusters around oocysts. (A) Midgut surface highlighting midgut progenitors in blood fed mosquitoes, 10 days post-feeding (one side of the midgut layer); 10.5 µm section. (B) Midgut surface highlighting midgut progenitors in *P. berghei* infected mosquitoes 10 days post-infection (one side of the midgut layer); 10.5 µm section. Midgut progenitors are shown in red and *Plasmodium* oocysts in green. Scale bar: 100 µm. Micrographs are representative of experiments that were independently reproduced at least 2 times with multiple individuals in each experimental group and obtained similar results.

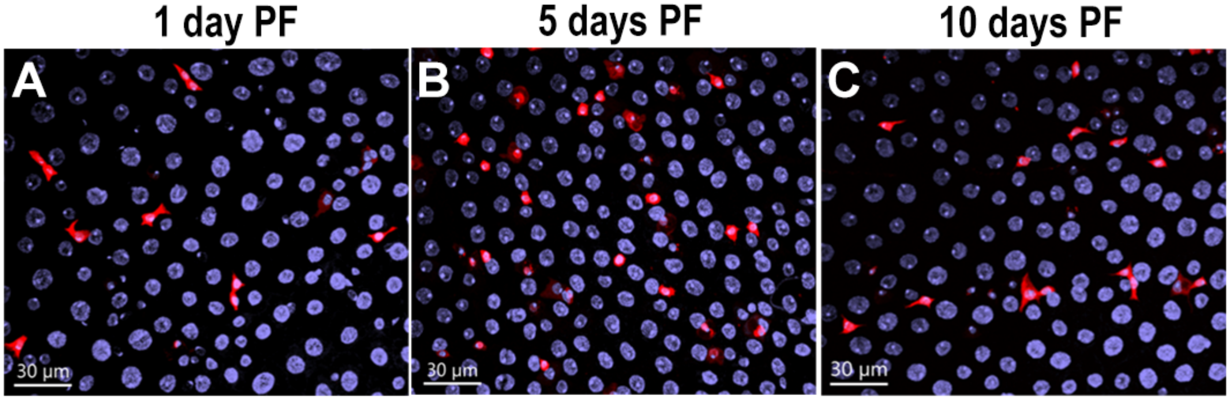

Supplementary Fig. 5. Midgut progenitors in blood fed mosquitoes at different time points after feeding. Midgut surface highlighting midgut progenitors in blood fed mosquitoes, (A) 1 day; (B) 5 days; and (C) 10 days post-feeding. Midgut progenitors are shown in red, and nuclei is in blue. Scale bar: 30 µm. Micrographs are representative of experiments that were independently reproduced at least 2 times with multiple individuals in each experimental group and obtained similar results.

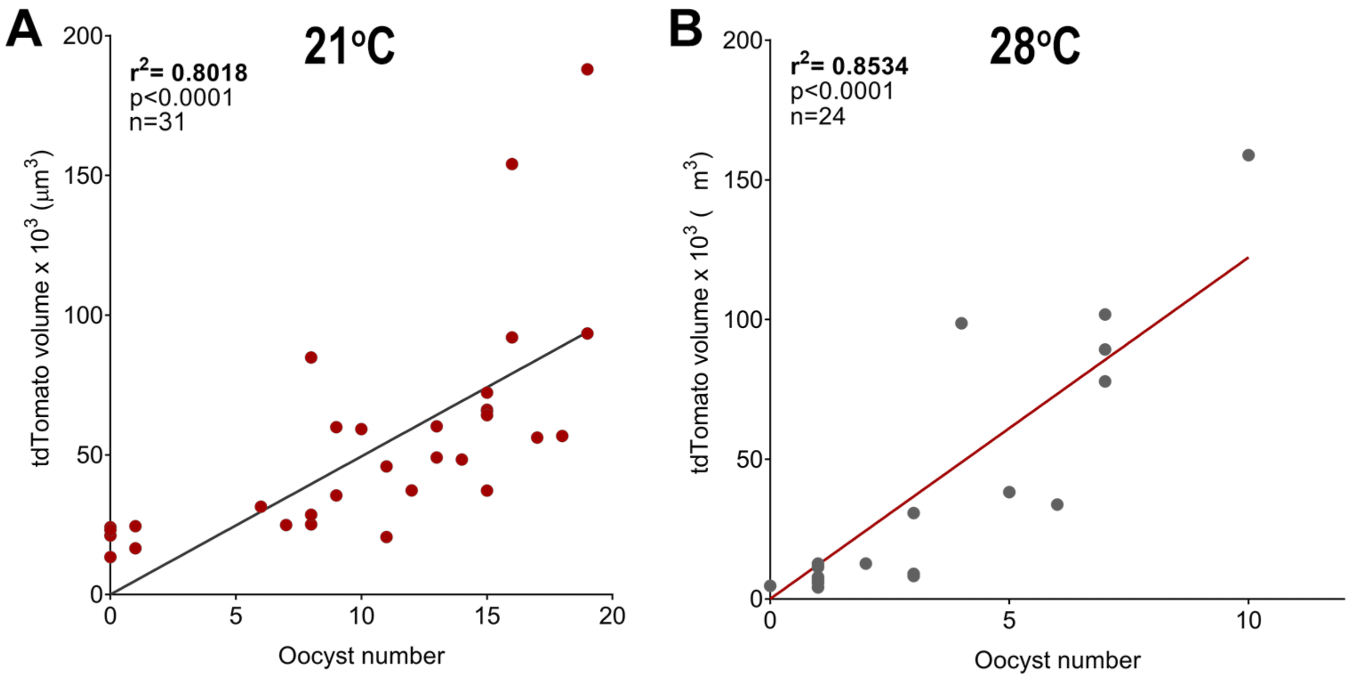

Supplementary Fig. 6. Midgut progenitor proliferation is dependent on the oocyst presence regardless of the temperature. (A) Correlation of oocyst numbers and tdTomato volume (tdTomato+ cells) of midguts from females kept at 21°C (r^2= 0.8018, p<0.0001, n=31) or (B) 28°C (r2= 0.8534, p<0.0001, n=24). Each dot represents an individual mosquito. Linear regression. Points correspond to the merge of two different experiments, from two independent infections. The numerical data underlying the plots in the manuscript are provided as a Source Data File.

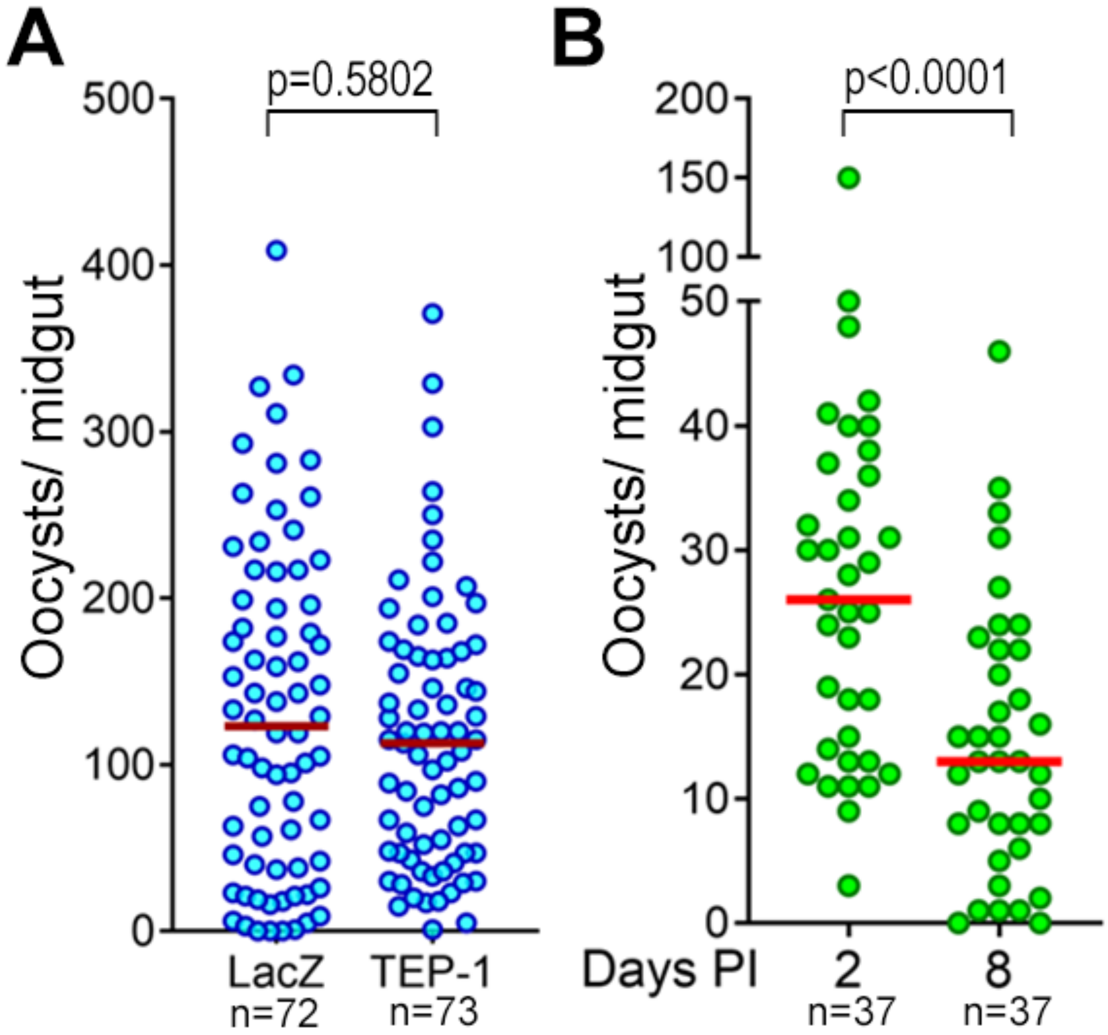

.

Supplementary Fig. 7. *Anopheles stephensi* eliminates oocysts using a late-phase immune mechanism. (A) Oocysts counts of lacZ and *TEP-1*-silenced midguts 10 days post *P.berghei* infection. (B) Oocysts counts of *An.stephensi* infected with *P.berghei* at day 2 and day 8 post-infection. Each dot represents an individual mosquito. Median is shown as red horizontal bars. Mann Whitney U test. The numerical data underlying the plots in the manuscript are provided as a Source Data File.

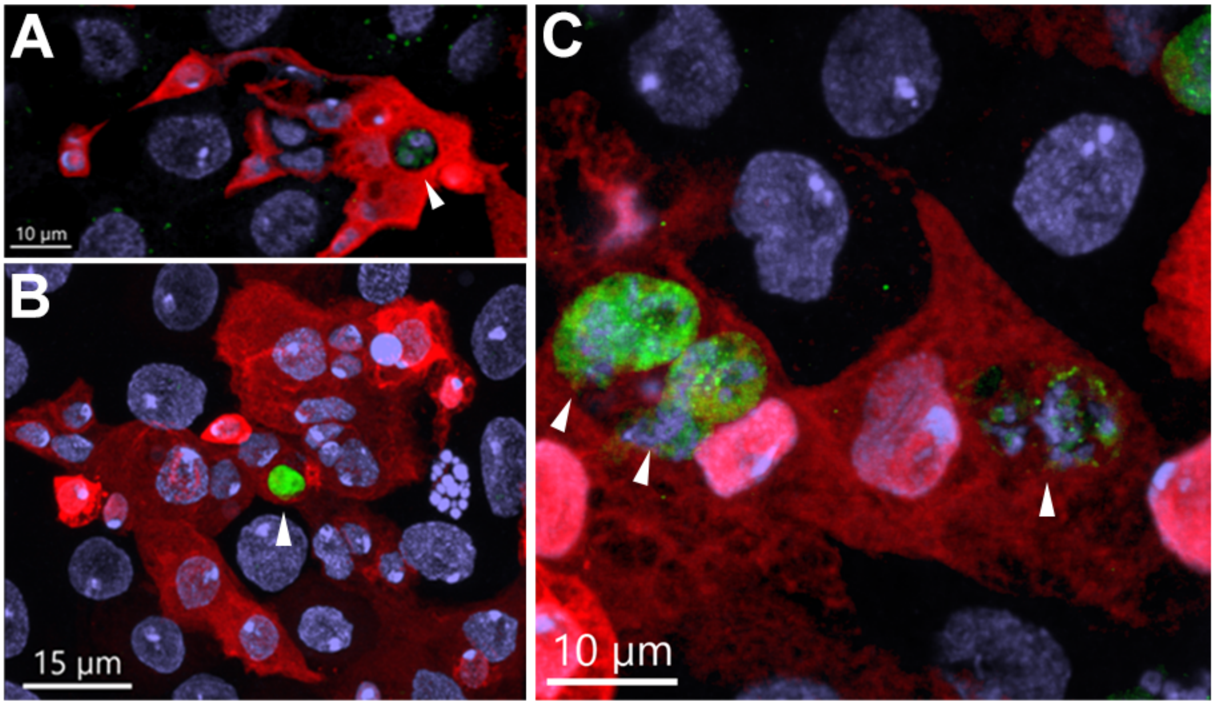

Supplementary Fig. 8. Midgut progenitors internalize oocysts fragments. (A) Oocyst found inside of a midgut progenitor. Oocyst nuclei are still visible. Scale Bar: 10 µm. (B) GFP from oocysts can be found inside midgut progenitors. Scale bar: 15 µm. (C) Close-up of pieces of oocysts inside midgut progenitors. Nuclei and GFP signal can be seen. Scale bar: 10 µm. White arrows indicate internalized parasites. Midgut progenitors are shown in red and *P.berghei* in green. Micrographs are representative of experiments that were independently reproduced at least 2 times with multiple individuals in each experimental group and obtained similar results.

Groups	Exp 1	Exp 2	Compiled	Percentage %
**Associated parasites - Live**	19	11	30	31
**Associated parasites - Dead**	43	23	66	69
**Total**	62	34	96	100

Supplementary Table 1. Percentage of dead oocysts associated with midgut progenitors in the midgut.
