# Supplementary material for: Mosquito midgut stem cell cellular defense response limits Plasmodium parasite infection
Source: Nat Commun. 2024 Feb 16;15:1422. doi: 10.1038/s41467-024-45550-2 (PMC10873411; doi:10.1038/s41467-024-45550-2)
Supplement: Supplementary file 3 — Description of Additional Supplementary Files [file 41467_2024_45550_MOESM3_ESM.pdf]

Description of Additional Supplementary Files

File Name: Supplementary Movie 1
Description: Midgut progenitors in direct contact eliminate *P.berghei* oocysts from the midgut epithelia 10 days after infection (Top view).

File Name: Supplementary Movie 2
Description: Midgut progenitors in direct contact eliminate *P.berghei* oocysts from the midgut epithelia 10 days after infection (Side view).

File Name: Supplementary Movie 3
Description: Midgut progenitors in direct contact eliminate *P.berghei* oocysts from the midgut epithelia 14 days after infection (Top view).

File Name: Supplementary Movie 4
Description: Midgut progenitors in direct contact eliminate *P.berghei* oocysts from the midgut epithelia 14 days after infection (Side view).
