# Supplementary material for: Mosquito midgut stem cell cellular defense response limits Plasmodium parasite infection
Source: Nat Commun. 2024 Feb 16;15:1422. doi: 10.1038/s41467-024-45550-2 (PMC10873411; doi:10.1038/s41467-024-45550-2)
Supplement: Supplementary file 8 — Reporting Summary [file 41467_2024_45550_MOESM8_ESM.pdf]

nature portfolio

Corresponding author(s): Carolina Barillas Mury, Ana Beatriz Barletta Ferreira

Last updated by author(s): November 7, 2023

Reporting Summary

Nature Portfolio wishes to improve the reproducibility of the work that we publish. This form provides structure for consistency and transparency in reporting. For further information on Nature Portfolio policies, see our Editorial Policies and the Editorial Policy Checklist.

Statistics

For all statistical analyses, confirm that the following items are present in the figure legend, table legend, main text, or Methods section.

n/a	Confirmed	
☐	☒	The exact sample size (*n*) for each experimental group/condition, given as a discrete number and unit of measurement
☐	☒	A statement on whether measurements were taken from distinct samples or whether the same sample was measured repeatedly
☐	☒	The statistical test(s) used AND whether they are one- or two-sided<br>*Only common tests should be described solely by name; describe more complex techniques in the Methods section.*
☒	☐	A description of all covariates tested
☒	☐	A description of any assumptions or corrections, such as tests of normality and adjustment for multiple comparisons
☐	☒	A full description of the statistical parameters including central tendency (e.g. means) or other basic estimates (e.g. regression coefficient) AND variation (e.g. standard deviation) or associated estimates of uncertainty (e.g. confidence intervals)
☐	☒	For null hypothesis testing, the test statistic (e.g. *F*, *t*, *r*) with confidence intervals, effect sizes, degrees of freedom and *P* value noted<br>*Give P values as exact values whenever suitable.*
☒	☐	For Bayesian analysis, information on the choice of priors and Markov chain Monte Carlo settings
☒	☐	For hierarchical and complex designs, identification of the appropriate level for tests and full reporting of outcomes
☒	☐	Estimates of effect sizes (e.g. Cohen's *d*, Pearson's *r*), indicating how they were calculated

Our web collection on statistics for biologists contains articles on many of the points above.

Software and code

Policy information about availability of computer code

Data collection	No software was used for that collection in this manuscript.
Data analysis	Prism 9 (GraphPad)

For manuscripts utilizing custom algorithms or software that are central to the research but not yet described in published literature, software must be made available to editors and reviewers. We strongly encourage code deposition in a community repository (e.g. GitHub). See the Nature Portfolio guidelines for submitting code & software for further information.

Data

Policy information about availability of data

All manuscripts must include a data availability statement. This statement should provide the following information, where applicable:

- Accession codes, unique identifiers, or web links for publicly available datasets
- A description of any restrictions on data availability
- For clinical datasets or third party data, please ensure that the statement adheres to our policy

All data supporting the findings of this study are available within the paper, its Supplementary Information and Source Data File.

Research involving human participants, their data, or biological material

Policy information about studies with human participants or human data. See also policy information about sex, gender (identity/presentation), and sexual orientation and race, ethnicity and racism.

Reporting on sex and gender	N/A
Reporting on race, ethnicity, or other socially relevant groupings	N/A
Population characteristics	N/A
Recruitment	N/A
Ethics oversight	N/A

Note that full information on the approval of the study protocol must also be provided in the manuscript.

Field-specific reporting

Please select the one below that is the best fit for your research. If you are not sure, read the appropriate sections before making your selection.

- [x] Life sciences
- [ ] Behavioural & social sciences
- [ ] Ecological, evolutionary & environmental sciences

For a reference copy of the document with all sections, see nature.com/documents/nr-reporting-summary-flat.pdf

Life sciences study design

All studies must disclose on these points even when the disclosure is negative.

Sample size	At least two independent experiments were performed for each assay. For gene expression experiments ate least three biological replicates per experimental group were considered per experiment. For infection studies, at least two infections were performed with at least 20 individuals per group. For imaging, each experiment contain at least 10 tissues per experimental group. Exact number of individuals for each experiment is detailed in the figure or legend. No software was used to determine sample size. Sample size was estimated based on previous studies using mosquito Plasmodium infections from our and other research groups.
Data exclusions	No data was excluded from the paper.
Replication	All experiments were replicate at least two times in independent experiments. Some of the assays were performed more than two times. All attempts resulted in similar results.
Randomization	Mosquito females were randomly assigned to control and experimental conditions.
Blinding	Researchers were aware of which individuals were part of the control and experimental groups.

Reporting for specific materials, systems and methods

We require information from authors about some types of materials, experimental systems and methods used in many studies. Here, indicate whether each material, system or method listed is relevant to your study. If you are not sure if a list item applies to your research, read the appropriate section before selecting a response.

Materials & experimental systems

n/a	Involved in the study	
☐	☒	Antibodies
☒	☐	Eukaryotic cell lines
☒	☐	Palaeontology and archaeology
☐	☒	Animals and other organisms
☒	☐	Clinical data
☒	☐	Dual use research of concern
☒	☐	Plants

Methods

n/a	Involved in the study	
☒	☐	ChIP-seq
☒	☐	Flow cytometry
☒	☐	MRI-based neuroimaging

Antibodies

Antibodies used	Living Colors® DsRed Polyclonal Antibody, cat# 632496, Takara Bio, San Jose, CA, USA;<br>Mouse monoclonal anti-RFP, Rocklands Immunochemicals, cat# 200-301-379, Philadelphia, PA, USA;<br>rabbit polyclonal anti-PbCap380 provided by Dr. Jacobs Lorena from Johns Hopkins School of Public Health (JHMI). Antibodies were produced and validated in house by rabbit immunization with the recombinant protein. Reference: Srinivasan P, Fujioka H, Jacobs-Lorena M. PbCap380, a novel oocyst capsule protein, is essential for malaria parasite survival in the mosquito. Cell Microbiol. 2008 Jun;10(6):1304-12. doi: 10.1111/j.1462-5822.2008.01127.x. Epub 2008 Feb 1. PMID: 18248630; PMCID: PMC4137771.<br>Anti-GFP antibody, cat# ab6556, Abcam, Cambridge, UK<br>mouse monoclonal Pbs21 provided by Dr. Jacobs Lorena from Johns Hopkins School of Public Health (JHMI). Antibodies were validated in the following publication: Winger LA, Tirawanchai N, Nicholas J, Carter HE, Smith JE, Sinden RE. Ookinete antigens of Plasmodium berghei. Appearance on the zygote surface of an mr 21 kD determinant identified by transmission-blocking monoclonal antibodies. Parasite Immunol. 1988;10:193–207.<br>Dl antibody (Delta), Cat # C594.9B, DSHB, Iowa City, IA, USA<br>Goat anti-rabbit Alexa 488 cat #A-11008, ThermoFisher Scientific, Waltham, MA, USA<br>Goat anti-rabbit Alexa 594 cat #A-11012, ThermoFisher Scientific, Waltham, MA, USA<br>Goat anti-mouse Alexa 488 cat #A-11001, ThermoFisher Scientific, Waltham, MA, USA<br>oat anti-mouse Alexa 594 cat #A-11005, ThermoFisher Scientific, Waltham, MA, USA
Validation	All commercial antibodies were validated by their manufacturers and were titrated in the lab to determine optimal concentration for experimentation.

Animals and other research organisms

Policy information about studies involving animals; ARRIVE guidelines recommended for reporting animal research, and Sex and Gender in Research

Laboratory animals	Three to 4-week-old female BALB/c mice obtained from Charles River, Wilmington, MA, USA. Mice were kept in individual ventilated cages at 20–21°C and 56% humidity and under a strict 12-hour light-dark cycle with sterile water and food provided ad libitum. Anopheles stephensi, HP10 strain (transgenic), females with between 4-7 days since emergence. HP10 mosquitoes was maintained as homozygotes at 28oC, 80% humidity under a 12h light/ dark cycle, and kept with 10% Karo syrup solution during adult stages.
Wild animals	No wild animals were used in this study.
Reporting on sex	Only female An.stephensi were used in this study because only females can transmit malaria due to blood feeding. Regarding mice infections, only female balb/c were used because they are more docile and fight less.
Field-collected samples	No field collected samples were used in this study.
Ethics oversight	Public Health Service Animal Welfare Assurance #A4149-01 guidelines were followed according to the National Institutes of Health Animal (NIH) Office of Animal Care and Use (OACU). These studies were done according to the NIH animal study protocol (ASP) approved by the NIH Animal Care and User Committee (ACUC), with approval ID ASP-LMVR5.

Note that full information on the approval of the study protocol must also be provided in the manuscript.

Plants

Seed stocks	N/A
Novel plant genotypes	N/A
Authentication	N/A
